# Magnetic Immunoassay Based on Au Pt Bimetallic Nanoparticles/Carbon Nanotube Hybrids for Sensitive Detection of Tetracycline Antibiotics

**DOI:** 10.3390/bios14070342

**Published:** 2024-07-15

**Authors:** Jianxia Lv, Rui Huang, Kun Zeng, Zhen Zhang

**Affiliations:** 1 National Narcotics Laboratory Beijing Regional Center, Beijing 100164, China; ljx19801128@sina.com; 2 School of Emergency Management, Jiangsu University, Zhenjiang 212013, China; 2222209005@ujs.edu.cn (R.H.); zhangzhen@ujs.edu.cn (Z.Z.); 3 School of the Environment and Safety Engineering, Jiangsu University, Zhenjiang 212013, China

**Keywords:** rapid detection, immunoassay, tetracycline, nanozyme

## Abstract

Misusage of tetracycline (TC) antibiotics residue in animal food has posed a significant threat to human health. Therefore, there is an urgent need to develop highly sensitive and robust assays for detecting TC. In the current study, gold and platinum nanoparticles were deposited on carbon nanotubes (CNTs) through the superposition method (Au@Pt/CNTs-s) and one-pot method (Au@Pt/CNTs-o). Au@Pt/CNTs-s displayed higher enzyme-like activity than Au@Pt/CNTs-o, which were utilized for the development of sensitive magnetic immunoassays. Under the optimized conditions, the limits of detection (LODs) of magnetic immunoassays assisted by Au@Pt/CNTs-s and Au@Pt/CNTs-o against TCs could reach 0.74 ng/mL and 1.74 ng/m, respectively, which were improved 6-fold and 2.5-fold in comparison with conventional magnetic immunoassay. In addition, the measurement of TC-family antibiotics was implemented by this assay, and ascribed to the antibody used that could recognize TC, oxytetracycline, chlortetracycline, and doxycycline with high cross-reactivity. Furthermore, the method showed good accuracy (recoveries, 92.1–114.5% for milk; 88.6–92.4% for pork samples), which also were applied for determination of the targets in real samples. This study provides novel insights into the rapid detection of targets based on high-performance nanocatalysts.

## 1. Introduction

Tetracycline (TC) antibiotics, including oxytetracycline (OTC), chlortetracycline (CTC), and doxycycline (DC), are widely used in clinical treatments and animal husbandry due to their broad-spectrum antibacterial effects [1,2,3]. However, TC can be discharged into the environment through medical wastewater, aquaculture wastewater, and livestock manure, which negatively impacts the ecological environment, such as by causing toxicity to microbial communities, and promoting drug resistance [4,5]. Additionally, TC can persist in animal-derived products through the food chain and pose health risks to humans via chronic toxicity, allergic symptoms, abnormal bone development, and even liver damage [6,7]. Thus, China has established the maximum residue limit of 100 μg/kg in milk and 200 μg/kg in muscle for TC, OTC, and CTC. For DC, the MRL is 100 μg/kg in muscle. Therefore, it is crucial to establish an accurate and sensitive detection method to screen the TC residues in food.

Traditional analytical techniques have been utilized to analyze TC antibiotics, including high-performance liquid chromatography (HPLC) [8,9], mass spectrometry (MS) [10,11], fluorescence methods [12], and immunoassays. However, instrumental methods and fluorescence methods have some disadvantages, including tedious operation, complicated sample preparations, and need for professional personnel [13]. Immunoassays are widely used in clinical testing, food safety, environmental monitoring, and other fields due to their high efficiency, low cost, and suitability for high-throughput screening characteristics [14,15]. Enzymes are the most commonly used signal probes due to their extremely strong catalytic activity. However, most biological enzymes, such as horse-radish peroxidase (HRP) and alkaline phosphatase (ALP), present some shortcomings, including poor stability, easy deactivation, and high cost, which could interfere with the performance of immunoassays [16]. The search for highly efficient and robust catalysts as a promising alternative to natural enzymes has become a popular research topic.

In recent years, nanomaterials with an inherent natural enzyme, named nanozymes, have attracted considerable attention. Since Gao et al. initially found that iron trioxide (Fe_3_O_4_) displayed peroxidase-like activity in 2007, more than 300 nanozymes have been reported to date [17,18,19,20]. Additionally, nanozymes were rated as one of “Top Ten Emerging Chemical Technologies” by the International Union of Pure and Applied Chemistry (IUPAC) in 2022. Compared with natural enzymes, nanozymes have advantages such as high stability, low preparation cost, and easy regulation of activity. Different types of novel nanozymes have been widely introduced in the biosensors [21,22], environment protection [23,24], and healthcare [25,26] fields in recent decades.

In the reported nanocatalysts, platinum (Pt) stands out as a highly promising nanocatalyst due to its exceptional activity for both hydrogen oxidation and oxygen reduction reactions (ORR) [27,28]. Considering the high cost, one potentially promising strategy is to produce a core–shell structure to expose the Pt layer on the surface, since only the outmost layer of the catalyst would participate in the reaction [29,30]. Pt-based multi-metallic NPs, such as AuPt [31] and PdPt [32] nanoparticles, have been extensively studied. These nanoparticles capitalize on the synergistic effect between metals, resulting in enhanced catalytic activity. In our research group, Au@Pt nanozyme was synthesized by a one-step method and showed superior catalytic efficiency compared to HRP [31].

Carbon nanotubes (CNTs) have attracted tremendous attention in electronics, energy, sensor, and others applications due to their large surface area, high conductivity, and low cost [33,34]. Many studies have focused on nanocatalysts supported by CNTs to enhance the catalytic behavior in the energy [34], sensor [35,36], and antibacterial [37] fields. Chen et al. designed a Pt-based nanozyme with ultra-thin Fe_2_O_3_ decoration in the inverse catalyst model with enhanced peroxidase activity and a colorimetric sensor for glucose determination based on 2Fe_2_O_3_/30Pt/CNTs, which improved almost 10 times in terms of sensitivity [36]. Cai et al. reported that CNTs-PdAu/Pt trimetallic nanoparticles exhibited an electrocatalytic peak current of up to 4.4 A mgPt^−1^ and high stability over 7000 s, which were far superior to Pt-based bimetallic NPs [38]. Inspired by these exciting results, designing nanocomposites that combine CNTs and Pt-based NPs holds great promise for creating high-performance catalysts as nanozyme labels.

In this study, gold and platinum nanoparticles were decorated on CNTs by the superposition method and one-pot method. The hybrid nanocatalysts were characterized and compared. Antibodies against TC were immobilized on magnetic beads orientation-ally via goat anti-mouse antibody as a linker. Novel immunomagnetic bead assays assisted by Au@Pt/CNTs were established to monitor the concentration of TCs in food. Milk samples and pork samples from a local market were collected and analyzed using the new method. This innovative approach promises to improve the monitoring and safety of food products by providing a sensitive and accurate detection method for TC.

## 2. Materials and Methods

### 2.1. Reagents and Materials

1-(3-(Dimethylamino)-propyl)-3-ethylcarbodiimide hydrochloride, N-hydroxysulosuccinimide (NHS), Tetramethylbenzidine (TMB), and goat anti-mouse antibody (GAM) were purchased from Sigma-Aldrich (St. Louis, MO, USA). The standards of TC, OTC, CTC, DC, Streptomycin (STR), chloramphenicol (CHL), cephalosporin (CEP), and enrofloxacin (ENR) were obtained from Beijing Zhongke Quality Inspection Biotechnology Co., Ltd. (Beijing, China). The monoclonal antibody against TC was obtained from Beijing WKHH Biotechnology Co., Ltd. (Beijing, China). Carboxylated MWCNTs (L = 5–30 μm) were purchased from Nanjing XFNANO Materials Tech Co., Ltd. (Nanjing, China). Other reagents were bought from Sinopharm Chemical Reagent Beijing Co., Ltd. (Beijing, China). Immunomagnetic beads (IMBs) were purchased from Suzhou Weidu Biological Co., Ltd. (Suzhou, China). Absorbance measurements were implemented with an M1000 PRO microplate reader by TECAN Inc. (Durham, NC, USA).

### 2.2. Development of OVA-TC and OVA-HRP

A quantity of 20 mg of PABA was dissolved in 4.5 mL of 0.2 mol/L HCl by stirring at 4 °C for 10 min. Then, 18 mg of NaNO_2_ in 0.5 mL of H_2_O was added dropwise. The reaction mixture was kept at 4 °C in the dark for 4 h to obtain solution A. Next, 5 mg of TC was dissolved in 3 mL of 0.05 mol/L ice-cold borax buffer solution, and 2 mL of solution A was added dropwise to this mixture. After mixing at 4 °C in the dark for 2 h, HBO_3_ was used to adjust the pH of the solution to 7.4. Subsequently, 10 mg of OVA or HRP, 10 mg of EDC, and 8 mg of NHS were added and stirred at room temperature for 6 h. The products, OVA-TC or HRP-TC, were purified by dialysis in PBS for 24 h, aliquoted, and stored at −20 °C.

### 2.3. Synthesis of Au@Pt/CNTs Nanohybrid Catalyst by Superposition Method

To begin, 58.32 mg of K_2_PtCl_6_, 49.44 mg of HAuCl_4_·4H_2_O, and 60 mg of Pluronic F127 were dissolved in 10 mL of deionized water. After thorough mixing, 105.6 mg of ascorbic acid was added, and the mixture was vortexed for 24 h at room temperature. The resulting mixture was centrifuged at 10,000 rpm for 20 min. The precipitate was then washed three times with water to remove any excess reagents. The collected product, Au@Pt, was dried at 37 °C for 24 h. To explore the enzyme activity of Au@Pt, the synthesis conditions were optimized by varying the amounts of K_2_PtCL_6_ and HAuCL_4_. The concentrations of K_2_PtCL_6_ were 0.4 mM, 2 mM, 10 mM, 20 mM, and 30 mM at 20 mM of HAuCL_4_, while the concentrations of HAuCL_4_ were 0.4 mM, 0.8 mM, 2 mM, 10 mM, 20 mM, and 50 mM at 30 mM of K_2_PtCL_6_.

Next, 0.2 mg of carboxylated MWCNTs was resuspended in 1 mL of MES buffer using ultrasonic agitation for 20 min. Subsequently, 50 μg of Au@Pt was added to the mixture and stirred at room temperature for 20 h. The resulting composite material, referred to as Au@Pt/CNTs-s, was centrifuged, washed twice with water, and dried at 37 °C for 24 h.

### 2.4. Synthesis of Au@Pt/CNTs Nanohybrid Catalyst by One-Pot Method

Quantities of 5.83 mg of K_2_PtCl_6_ solution, 4.94 mg of HAuCl_4_.4H_2_O, 6 mg of Pluronic F127, and 0.2 mg of carboxylated MWCNTs were mixed in 2 mL of ultrapure water. To this mixture, 20 mg of ascorbic acid in 1 mL of ultrapure water was added dropwise, followed by sonication for 15 min. Next, the mixture was stirred magnetically at room temperature for 24 h. The obtained product, Au@Pt/CNTs-o, was washed with ultrapure water and separated by centrifugation at 10,000 rpm for 20 min.

### 2.5. Characteristic of Au@Pt/CNTs Nanohybrid

TEM images were obtained using a Tecnai G2 spirit BioTwin (FEI, Hillsborough, OR, USA) electron microscope operated at 120 kV. To monitor the enzyme-like property of the Au@Pt/CNTs nanohybrid, TMB/H_2_O_2_ solution in acetate buffer solution was utilized to indicate the color change.

### 2.6. Immobilization of GAM on IMBs

Quantities of 100 μL IMB, 10 mg EDC, and 10 mg NHS were mixed in 1 mL MES buffer for 2 h at 4 °C. By the aid of magnetism, activated IMB was removed from excess EDC and NHS, and washed with MES buffer twice. Then, an amount of GAM was reacted with the activated IMB with stirring for 12 h at 4 °C. After the magnetic separation and washing step, IMB-GAM was obtained and stored in MES buffer containing 2% BSA.

### 2.7. Combination of Color Probe Based on Au@Pt/CNTs Nanohybrid

A quantity of 200 μL of Au@Pt/CNTs-s or Au@Pt/CNTs-o obtained was added with 15 μL of OVA-TC, and stirred at 4 °C for 2 h. After centrifuging at 5000 rpm for 10 min, the pellet was washed by PBST twice and then the products were resuspended in 1 mL of PBS (containing 2% BSA) solution at 4 °C for later use.

### 2.8. Establishment of Magnetic Immunoassay Assisted by Au@Pt/CNTs Nanohybrid

Quantities of 50 μL of IMB-GAM in PBS containing 0.1% BSA and 0.01% Tween-20, 50 μL of TC antibody, 50 μL of Au@Pt/CNTs-o/OVA-TC or Au@Pt/CNTs-s/OVA-TC, and 50 μL of TC standards were mixed together and reacted at 37 °C for 30 min. By the aid of the magnetic separation, the supernatant was discarded and the participates was washed by PBS thrice. Then, 200 mL of freshly prepared TMB/H_2_O_2_ was adopted and incubated at 37 °C for 10 min. After adding 100 mL of 2 mol/L H_2_SO_4_, the supernatants absorbed by magnet were transferred onto a 96-well plate to test the absorbance value at 450 nm.

### 2.9. Optimization of Magnetic Immunoassay Assisted by Au@Pt/CNTs

Virous reaction conditions may interfere with the performance of immunoassay. In sample buffer, several parameters were estimated, including pH (5.0, 6.0, 7.0, 7.4, 8.0, and 9.0), the concentrations of Na^+^ (0, 0.005, 0.01, 0.05, 0.1, and 1 mol/L), acetonitrile (0, 5%, 10%, 20%, 30%, and 50%, *m*/*v*), and unrelated protein (0%, 0.5%, 1%, 2.5%, 5%, and 10%, *m*/*v*). The maximum absorbances (B0) were recorded and IC50 was the concentration at which 50% of the antibodies were bound to the analyte.

### 2.10. Sample Preparation and Analysis

Milk samples and pork samples were collected from local markets in Zhenjiang, China. Each 5 mL milk sample was mixed with 5 mL of 1% (*v*/*v*) trichloroacetic acid, and the mixture was ultrasonicated for 30 min at room temperature. After centrifugation at 5000× *g* for 10 min, the supernatant was collected and filtered through 0.45 µm membranes. To mitigate the matrix effect from the milk, the supernatant was diluted before the detection process. For the pork samples, we followed the pretreatment method specified in the “National Standard of the People’s Republic of China” (GB 31658.6-2021) [39]. Briefly, 5 g of the homogenized pork sample was mixed with 20 mL of EDTA.2Na-Mcllvaine buffer solution and shaken for 10 min. Subsequently, 5 mL of sulfuric acid solution and 5 mL of sodium tungstate solution were added. After vortexing for 5 min, the mixture was centrifuged at 5000× *g* for 10 min. The supernatant was then filtered using neutral filter paper and prepared for analysis.

To evaluate the recovery rate of the new method, a certain concentration of TCs was spiked in milk and pork samples. Following the preparation process above, the recovery rate was calculated. Eight commercial milk samples and eight pork samples from a local dairy farm were collected and determined by magnetic immunoassay assisted by Au@Pt/CNTs and UHPLC/MS.

## 3. Results

### 3.1. Construction, Optimization, and Characterization of Au@Pt/CNTs Nanohybrid

**Figure 1:**
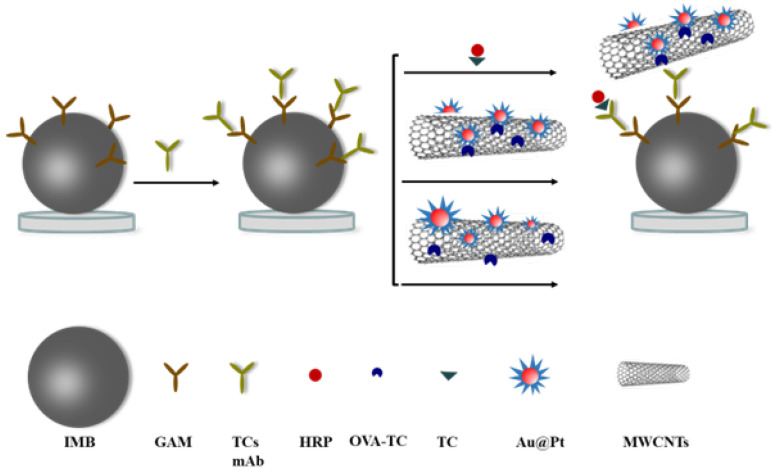
The diagram of magnetic immunoassay assisted by Au@Pt/CNTs.

To explore the effect of the synthesis on the catalytic activity of nanohybrids, two approaches were adopted. For Au@Pt/CNTs-s, Au@Pt developed were precipitated on CNTs, while for Au@Pt/CNTs-o, AuNPs and Pt nanoparticles were directly reduced onto CNTs (Figure 1). To enhance the enzyme activity of Au@Pt/CNTs-s, the synthesis conditions were optimized by varying the amounts of K_2_PtCL_6_ and HAuCL_4_ first (Figures S1–S3). As seen in Figure S1, only gold nanoparticles were present when the concentration of K_2_PtCl_6_ was 0.4 mM and 2 mM. With increasing K_2_PtCl_6_ concentration, Pt gradually recombined on the surface of gold nanoparticles, forming a dandelion-like structure. The catalytical results indicated a positive correlation between absorbance and K_2_PtCl_6_ concentration (Figure S3a). When the concentration of HAuCl4 ranged from 0.8 mM to 20 mM, the size of the composite particles decreased significantly, but increased sharply at 50 mM HAuCl4 (Figure S2). The maximum absorbance was observed at 2 mM HAuCl_4_ (Figure S3b). Therefore, the optimal conditions were determined to be 30 mM K_2_PtCl_6_ and 2 mM HAuCl_4_, and the average diameter was 54 nm for the obtained Au@Pt particles (Figure S4).

**Figure 2:**
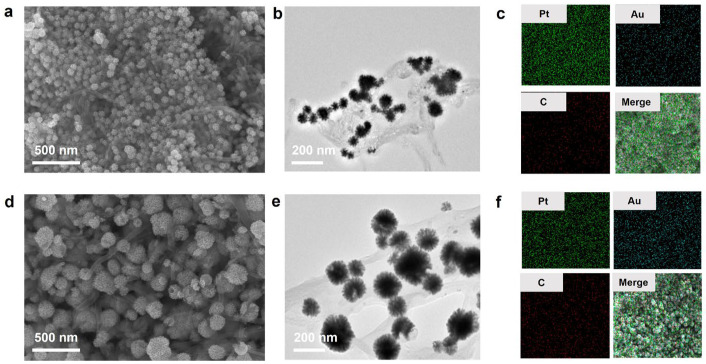
Characterizations of Au@Pt/CNTs nanohybrid. (**a**–**c**) SEM, TEM, and EDS images of Au@Pt/CNTs-s; (**d**–**f**) SEM, TEM, and EDS images of Au@Pt/CNTs-o.

Next, Au@Pt nanoparticles were assembled on MWCNTs. SEM and TEM images revealed various dandelion-shaped nanoparticles embedded on the surface of CNTs [Figure 2a,b,d,e]. For Au@Pt/CNTs-s, uniformly sized Au@Pt particles with an average diameter of about 50 nm covered the CNTs. In contrast, Au@Pt/CNTs-o exhibited Au@Pt particles with diameters ranging from 20 nm to 200 nm. It is speculated that the presence of CNTs in the solution may have hindered the reduction reaction of K_2_PtCl_6_ and HAuCl_4_, leading to variations in reagent concentrations in local areas and resulting in differently sized Au@Pt particles. SEM/EDS element maps of these two hybrid nanomaterials showed that the Au@Pt particles were uniformly decorated on the surface of CNTs for both nanohybrids and the Pt content in Au@Pt/CNTs-s was significantly higher than that in Au@Pt/CNTs-o [Figure 2c,f].

### 3.2. Enzyme Mimetic Activity of Au@Pt/CNTs Nanohybrid

**Figure 3:**
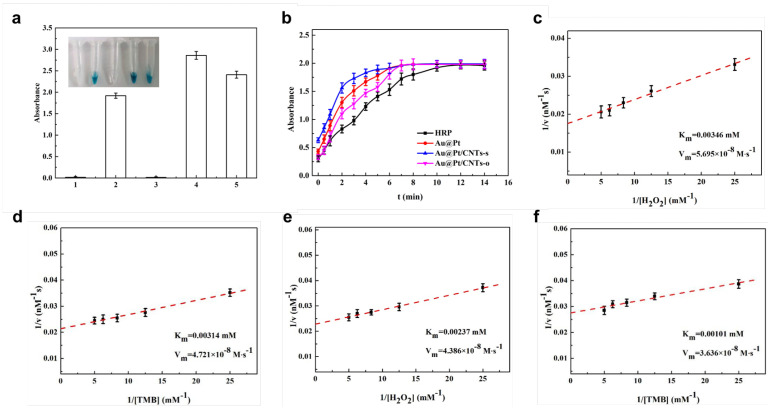
(**a**) Identification of enzyme−like probes: 1 uncarboxylated MWCNTs; 2 Au@Pt; 3 carboxylated MWCNTs; 4 Au@Pt/CNTs-s; 5 Au@Pt/CNTs-o. (**b**) Kinetic curve of enzymatic reaction; the double reciprocal plot of the activity of Au@Pt/CNTs-s (**c**,**d**) and Au@Pt/CNTs-o (**e**,**f**).

Further, the peroxidase-like activities of these nanomaterials were evaluated with the help of TMB/H_2_O_2_ solution (Figure 3). MWCNTs and carboxylated MWCNTs did not catalyze TMB solution to turn blue, whereas Au@Pt, Au@Pt/CNTs-s, and Au@Pt/CNTs-o displayed significant peroxidase-like activity (Figure 3a). To investigate the enzymatic kinetic curves, the catalysts were added to 100 μL TMB chromogenic solution and the absorbance values were measured at 30 s intervals. As seen from Figure 3b, the absorbance of Au@Pt/CNTs-s began to level out after 7 min, while that of Au@Pt and Au@Pt/CNTs-o plateaued after 8 min. In contrast, HRP’s absorbance leveled out after 12 min. These results indicated that Au@Pt/CNTs-s exhibited the highest enzymatic reaction rate.

The apparent kinetic parameters of these catalysts were calculated from the double reciprocal of the Michaelis–Menten equation (Figure 3c–f). Since the higher Km indicates the lower affinity towards the substrate, Au@Pt/CNTs-s and Au@Pt/CNTs-o showed higher affinity with TMB and H_2_O_2_ compared with HRP and other Pt-based nanohybrids, including GOCNT-Pt [35], CNT-Pt [35], 2Fe_2_O_3_/30Pt/CNTs [36], and30Pt/CNTs [37]. In comparison with other nanohybrid catalysts (Table 1), Au@Pt/CNTs-s and Au@Pt/CNTs-o displayed the highest affinity. Additionally, it was found the catalytical activity of Au@Pt/CNTs-s was higher than that of Au@Pt/CNTs-o. Since Au@Pt nanoparticles played an essential role in peroxidase-like activities of these two materials, it was believed that the variation in Au@Pt nanoparticle size in Au@Pt/CNTs-o might have led to a reduction in catalytic efficiency compared to Au@Pt/CNTs-s.

### 3.3. Optimization of Magnetic Immunoassay Assisted by Au@Pt/CNTs

**Figure 4:**
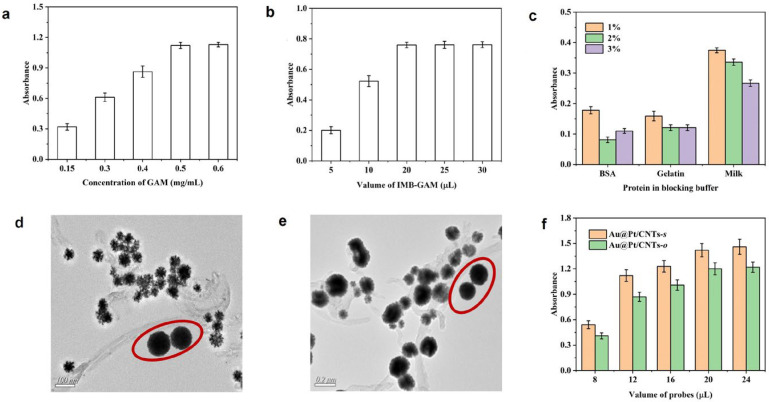
Optimization of reaction parameters: (**a**) concentration of GAM; (**b**) volume of IMB-GAM; (**c**) blocking buffer. TEM images of combination between IMB and Au@Pt/CNTs-s (**d**) or Au@Pt/CNTs-o (**e**); IMBs are circled in red. Optimization of the volume of probes (**f**).

To capture the trace target in samples, it is essential to first develop specific anti-body-decorated IMBs. In this study, GAM was utilized as a linker to immobilize antibodies in an oriented manner through their Fc fragments. The synthesis conditions for IMB-GAM were optimized, including the concentration of GAM, the amount of IMB-GAM used, and the choice of blocking reagents. The absorbance increased with the rising concentration of GAM, reaching a plateau beyond 0.5 mg/mL GAM. The maximum absorbance was achieved when 20 mL of IMB-GAM was used (Figure 4a,b), thus identifying the optimal conditions. Appropriate blocking conditions are crucial to reduce background interference and enhance the method’s performance. Various reagents, including BSA, gelatin, and skimmed milk powder, were tested at different concentrations. It was found that 2% BSA provided the lowest background interference, indicating it to be the most effective blocking reagent (Figure 4c).

To obtain the chromogenic probe, Au@Pt/CNTs were linked with OVA-TC by physical absorbance. After reacting with IMB-GAM, it was observed by TEM that these nanohybrids were tightly combined with IMB (Figure 4d,e), which indicated the successful development of the chromogenic probe. Next, the usages of the chromogenic probe were optimized, as shown in Figure 4f. The absorbance enhanced with the increasing volume of the probe. At 20 μL of Au@Pt/MWCNTs-s and 20 μL of Au@Pt/MWCNTs-o, the value reached the platform, which was applied in further tests.

**Figure 5:**
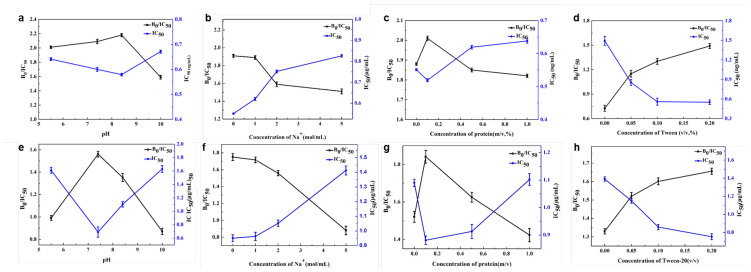
Optimal conditions in magnetic immunoassay assisted by Au@Pt/CNTs-s (**a**–**d**) and Au@Pt/CNTs-o (**e**–**h**).

The physical parameters in the immunoassay were evaluated, including Na+ concentration, pH, protein concentration, and Tween-20. As shown in Figure 5, the optimal pH was identified as 8.4 for Au@Pt/CNTs-s and 7.4 for Au@Pt/CNTs-o, as these conditions yielded the maximum B0/IC50 and the lowest IC50. The presence of Na+ resulted in a decline in B0/IC50 for both immunoassays based on Au@Pt/CNTs-s and Au@Pt/CNTs-o, indicating decreased sensitivity. Adding a certain amount of unrelated protein could reduce nonspecific binding and background noise. The results demonstrated that 0.1% BSA in the buffer achieved the highest B0/IC50 for both magnetic immunoassays, corresponding to the highest sensitivity. In our experiment, we observed that these nanohybrids tended to adsorb to polystyrene, resulting in higher background levels. To improve the dispersion of enzyme-like materials, surfactants such as Tween-20 were added. A concentration of 0.2% Tween-20 in the buffer provided the best performance for both magnetic immunoassays.

### 3.4. Analytical Performance of Magnetic Immunoassays Assisted by Au@Pt/CNTs

**Figure 6:**
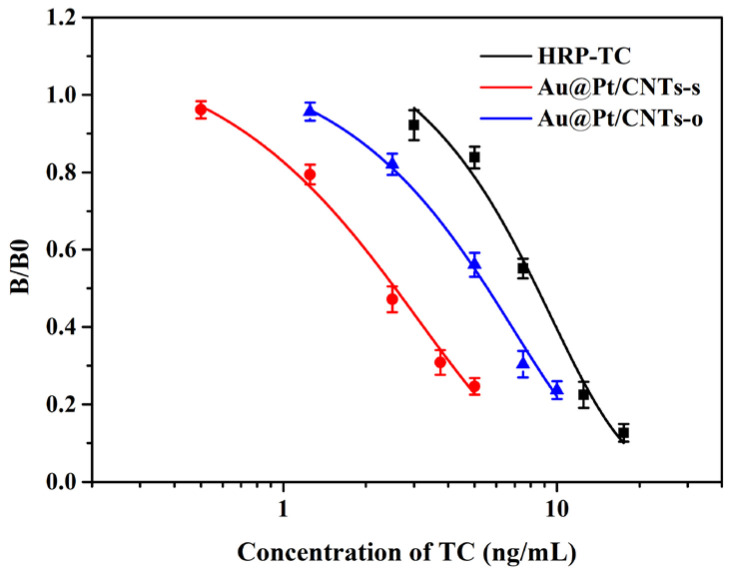
Standard curves of magnetic immunoassays assisted by HRP-TC, Au@Pt/CNTs-s, and Au@Pt/CNTs-o.

Based on these optimal conditions, we developed magnetic immunoassays assisted by Au@Pt/CNTs nanohybrids. The standard curves, obtained through a four-parameter fitting, are depicted in Figure 6. Notably, the curves of magnetic immunoassays assisted by Au@Pt/CNTs exhibited a significant leftward shift compared to conventional magnetic immunoassays, indicating enhanced sensitivity. The limits of detection (LODs) for magnetic immunoassays based on Au@Pt/CNTs-s, Au@Pt/CNTs-o, and HRP-TC were determined to be 0.74 ng/mL, 1.74 ng/mL, and 4.25 ng/mL, respectively. These corresponded to detection ranges of 1.10–6.57 ng/mL, 2.53–12.34 ng/mL, and 4.85–15.25 ng/mL, respectively. Moreover, the magnetic immunoassays based on Au@Pt/CNTs-s and Au@Pt/CNTs-o exhibited a 6-fold and 2.5-fold improvement in sensitivity, respectively, compared to conventional magnetic immunoassays.

### 3.5. Analysis of Samples

**Figure 7:**
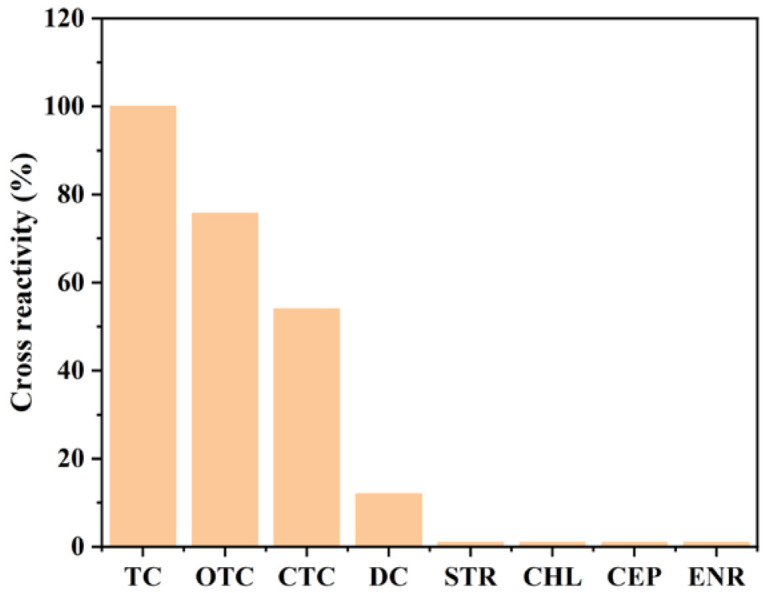
Cross-reactivity of magnetic immunoassays assisted by Au@Pt/CNTs-s.

The proposed magnetic immunoassay assisted by Au@Pt/CNTs-s was utilized to determine the concentration of TC in real samples. To ensure the specificity of the assay, its cross-reactivity was assessed. The results demonstrated that this assay could accurately analyze TC-family antibiotics with the cross-reactivity of 75.7%, 54%, and 12% for OTC, CTC, and DC, respectively. Additionally, no cross-reactivity was observed with other antibiotics, such as STR, CHL, CEP, and ENR (Figure 7). Further, to evaluate the applicability of the prepared sensor for practical applications, recovery rates were tested by spiking TC into milk and pork samples. The recovery rates ranged from 92.1% to 114.5% for milk and from 88.6% to 92.4% for pork (Table 2), indicating that the magnetic immunoassay assisted by Au@Pt/CNTs-s is a reliable tool for TC detection in real samples. Most of the samples did not contain detectable levels of TCs, except for sample No. 7 in milk, which had 1.05 ng/mL, and sample No. 5 in pork, which had 5.57 ng/mL of TCs (Table 3). The detection rates were 87.5% for both milk and pork samples, and all collected samples met the maximum residue limit (MRL) standards in China.

## 4. Discussion

Nanocatalysts have garnered increasing interest due to their high and robust catalytic activity, low cost, and good stability under harsh conditions. Pt-based nanozymes are among the most efficient catalytic materials, owing to their high enzyme-like activity. However, the high cost of Pt and the kinetic limitations of its oxygen reduction reactions have hindered broader applications. To minimize Pt consumption and optimize catalytic performance, significant efforts have been directed towards synthesizing Pt-based multi-metallic nanoparticles and supporting Pt on nanomaterials with high specific surface areas. In the realm of Pt-based multi-metallic NPs, combinations such as AuPt [31], PdPt NPs [32], and PdAu/Pt [38] have been extensively explored. These nanozymes demonstrate enhanced performance due to the possible synergistic effects between the metals. Another effective strategy involves using carbon-based materials, like CNTs or graphene, as supports. For example, Yang et al. reported that small Pt nanoparticles (0.55–2.81 nm) were loaded on CNTs via atomic layer deposition, and 30Pt/CNTs nanozymes with a size of 1.69 nm exhibited significantly higher peroxidase-like activity [36]. In this study, we combined both strategies by supporting Au@Pt core–shell nanoparticles on CNTs using the superposition method and the one-pot method. The resulting hybrids, particularly Au@Pt/CNTs-s, exhibited higher activity compared to GOCNT-Pt [35], CNT-Pt [35], 30Pt/CNTs [36], and 2Fe_2_O_3_/30Pt/CNTs [37]. This confirms that integrating these approaches is a promising strategy for designing and synthesizing effective nanocatalysts. Additionally, for both Au@Pt/CNTs-s and Au@Pt/CNTs-o, Au@Pt particles were uniformly immobilized on CNTs, which would help to maximize the utilization efficiency of active sites, thereby improving the efficiency and selectivity of catalytic reactions [40,41]. Furthermore, it was reported that AuPt bimetallic nanoparticles presented a plasmonic effect [42,43]. To avoid the interfere of this feature on absorbance, the supernatants without any nanomaterials were collected and tested.

Detection methods integrated with various nanozymes have been explored for TC in recent years (Table 4). Electrochemical and colorimetric sensors have been established using aptamers [44] or molecular imprinting polymers (MIP) [45] as capture elements. Additionally, multi-mode assays have been developed and evaluated, leveraging the diverse characteristics of nanozymes. For instance, Gogoi et al. prepared novel borophene quantum dots (QDs) for the colorimetric detection of TC, achieving a limit of detection (LOD) of 1.02 μM [46]. Furthermore, the fluorescence of borophene QDs was quenched by TC through the inner filter effect mechanism, resulting in a fluorescence sensor with an LOD of 1.08 μM. In comparison with the listed methods, the methods developed in this study exhibited the better performance. Additionally, it was observed that those magnetic immunoassays assisted by Au@Pt/CNTs demonstrated improved sensitivity compared with conventional magnetic immunoassays, with LODs 6-fold and 2.5-fold higher for Au@Pt/CNTs-s and Au@Pt/CNTs-o. Magnetic immunoassays assisted by Au@Pt/CNTs-s showed better sensitivity than those using Au@Pt/CNTs-o, which may be attributed to the higher catalytic activity of Au@Pt/CNTs-s.

Furthermore, it was noticed that this novel assay could recognize the TC family of antibiotics, while most of the methods listed presented specific binding with TC. It was speculated that the antibody used in this study displayed class specificity towards TC analogs. Due to the similar structure of TCs, some antibodies can bind more than one TC and consistent results have been reported. For example, Chen et al. established an ELISA based on a broad-spectrum monoclonal antibody with the IC50 of 0.72 ng/mL for TC, 3.2 ng/mL for OTC, and 6.4 ng/mL for CTC [47]. According to the government regulations, the total tetracycline residues, including TC, OTC, CTC, and DC, need to be monitored in animal-origin food. Therefore, this study provides an efficient and convenient tool for the evaluation of TC pollution in food.

**Table 4:** Nanoparticle-based sensors for the detection of TCs.

Nanoparticles	Biosensors	LOD	Targets	Ref.
Ni^2+^-2,3,6,7,10,11-hexahydroxytriphenylene (Ni-HHTP)	electrochemical aptasensor	1.9 pM	TC	[44]
Fe_3_O_4_ @MIP	Colorimetric biosensor	0.4 μM	TC	[45]
borophene quantum dots (QDs)	Colorimetric biosensors	1.02 μM	OTC, TC	[46]
	Fluorescent biosensors	1.08 μM	OTC, TC	
non-spherical gold nanoparticle/black phosphorus nanocomposite (BP-nsAu NPs)	Colorimetric biosensor	90 nM	TC	[48]
NH_2_-MIL-88 B (Fe, Ni)	Colorimetric biosensors	0.182 μM	TC, OTC, CTC, DC	[49]
	Fluorescent biosensors	0.0668 μM	TC, OTC, CTC, DC	
Au@Pt/CNTs-s	Colorimetric immunoassay	0.74 ng/mL	TC, OTC, CTC, DC	This work
Au@Pt/CNTs-o	Colorimetric immunoassay	1.74 ng/mL	TC, OTC, CTC, DC	This work

## 5. Conclusions

In this study, Au@Pt nanoparticles were decorated on the surface of CNTs using two different strategies. Au@Pt/CNTs-s exhibited higher enzyme-like activity compared to Au@Pt/CNTs-o and other Pt-based nanocatalysts. Magnetic immunoassays assisted by Au@Pt/CNTs were established and compared, with the method assisted by Au@Pt/CNTs-s showing the highest performance, achieving a limit of detection (LOD) of 0.74 ng/mL. This novel assay could recognize TC-family antibiotics, including TC, OTC, CTC, and DC. The recovery rates were 92.1–114.5% for milk and 88.6–92.4% for pork samples. In real samples, the concentrations of TCs ranged from ND to 1.05 ng/mL in milk, and from ND to 5.57 ng/mL in pork. This study highlights that Au@Pt/CNTs nanoparticles can offer novel strategies for mimic-enzyme labels in biosensing for food safety.

## Figures and Tables

**Table 1:** Apparent kinetic parameters of different Pt-based catalysts and HRP.

Catalyst	Substrate	Km (mM)	Vm (10^−8^ Ms^−1^)	Reference
HRP	TMB	0.329	7.64	[31]
	H_2_O_2_	1.468	22.88	
Au@Pt	TMB	2.431 × 10^−3^	4.43	[31]
	H_2_O_2_	4.08 × 10^−3^	6.01	
Au@Pt/CNTs-s	TMB	1.01 × 10^−3^	3.63	This work
	H_2_O_2_	2.37 × 10^−3^	4.39	
Au@Pt/CNTs-o	TMB	3.14 × 10^−3^	4.72	This work
	H_2_O_2_	3.46 × 10^−3^	5.69	
GOCNT-Pt	TMB	0.075	0.302	[35]
	H_2_O_2_	1.82	1.27	
CNT-Pt	TMB	0.152	0.422	[35]
	H_2_O_2_	6.24	2.18	
2Fe_2_O_3_/30Pt/CNTs	TMB	0.17	18.1	[36]
	H_2_O_2_	0.053	6.79	
30Pt/CNTs	TMB	1.15	18.9	[37]
	H_2_O_2_	72.91	103	

**Table 2:** Recovery and precision of the established method (*n* = 4).

	Spiked Concentration (ng/mL)	Found Concentration (ng/mL)	Recovery Rate	CV (%)
Milk	0	<LOD	−	−
	2	2.28 ± 0.08	114.5%	7.8%
	5	5.32 ± 0.43	106.4%	10.9%
	10	9.21 ± 1.39	92.1%	11.5%
pork	0	<LOD	−	−
	2	1.79 ± 0.23	89.5%	11.9%
	5	4.43 ± 0.56	88.6%	11.6%
	10	9.24 ± 1.34	92.4%	10.5%

**Table 3:** Measurement of TCs in milk and pork samples.

Sample Number	Results of Milk Samples (ng/mL)		Results of Pork Samples (ng/mL)	
	By the Established Method	By UPLC-MS/MS	By the Established Method	By UPLC-MS/MS
1	ND ^1^	ND	ND	ND
2	ND	ND	ND	ND
3	ND	ND	ND	ND
4	ND	ND	ND	ND
5	ND	ND	5.57	6.78
6	ND	ND	ND	ND
7	1.05	2.15	ND	ND
8	ND	ND	ND	1.43

^1^ ND: not detected.

## Data Availability

The data that support the findings of this study are available upon reasonable request.

## Supplementary Materials

The following supporting information can be downloaded at: https://www.mdpi.com/article/10.3390/bios14070342/s1, Figure S1. TEM images of Au@Pt using 20 mM HAuCl_4_ and certain concentration of K_2_PtCL_6_. Figure S2. TEM images of Au@Pt using 30 mM K_2_PtCL_6_ and certain concentration of HAuCl_4_. Figure S3. Absorbance of Au@Pt at different concentrations of K_2_PtCL_6_ and HAuCl_4_. Figure S4. DLS analysis of Au@Pt at optimal conditions.
