# Supplementary material for: Magnetic Immunoassay Based on Au Pt Bimetallic Nanoparticles/Carbon Nanotube Hybrids for Sensitive Detection of Tetracycline Antibiotics
Source: Biosensors (Basel). 2024 Jul 15;14(7):342. doi: 10.3390/bios14070342 (PMC11274607; doi:10.3390/bios14070342)
Supplement: Supplementary file 1 [file biosensors-14-00342-s001.zip › biosensors-3055870-supplementary.pdf]

Supplementary materials

Magnetic Immunoassay Based on Au Pt Bimetallic Nanoparticles/Carbon Nanotube Hybrids for Sensitive Detection of Tetracycline Antibiotics

Jianxia Lv [1], Rui Huang [2], Kun Zeng [2,*] and Zhen Zhang [2]

[1] National Narcotics Laboratory Beijing Regional Center, Beijing 100164, China, ljx19801128@sina.com
[2] School of Emergency Management, School of the Environment and Safety Engineering, Jiangsu University, Zhenjiang 212013, China, 2222209005@ujs.edu.cn (R.H.); zhangzhen@ujs.edu.cn (Z.Z.)
* Correspondence: kjj80116@ujs.edu.cn

Figure S1. TEM images of Au@Pt using 20mM $HAuCl_4$ and certain concentration of K_2PtCL_6.

Figure S2. TEM images of Au@Pt using 30mM K_2PtCL_6 and certain concentration of $HAuCl_4$.

Figure S3. Absorbance of Au@Pt at different concentration of K_2PtCL_6 and $HAuCl_4$.

Figure S4. DLS analysis of Au@Pt at optimal condition.

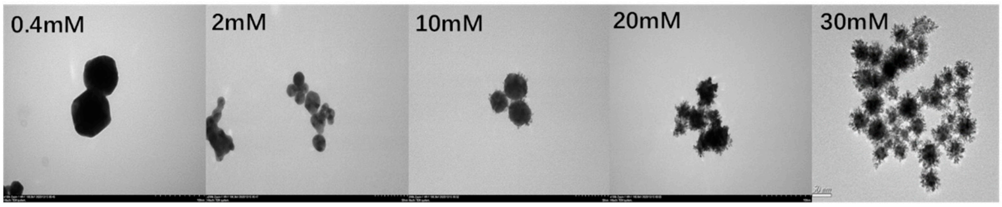

Figure S1. TEM images of Au@Pt using 20mM $HAuCl_4$ and certain concentration of K_2PtCL_6.

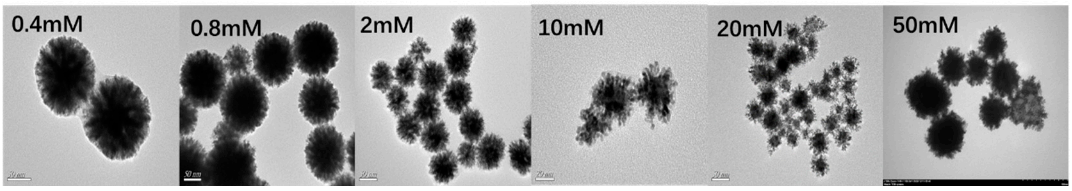

Figure S2. TEM images of Au@Pt using 30mM K_2PtCL_6 and certain concentration of $HAuCl_4$.

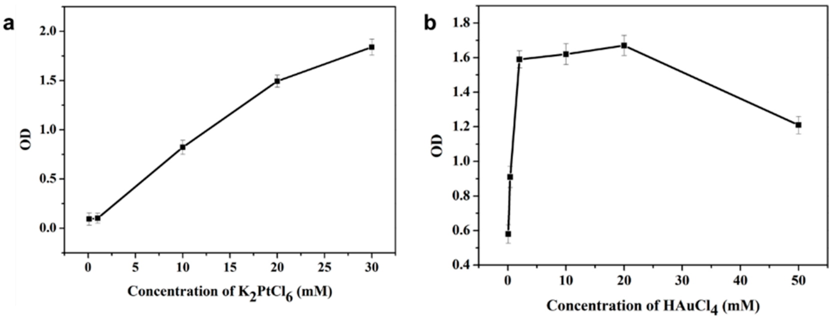

Figure S3. Absorbance of Au@Pt at different concentration of K_2PtCL_6 and $HAuCl_4$. a: The concentrations of K_2PtCL_6 were 0.4 mM, 2 mM, 10 mM, 20 mM and 30 mM at 20 mM of $HAuCL_4$; b: the concentrations of $HAuCL_4$ were 0.4 mM, 0.8 mM, 2 mM, 10 mM, 20 mM and 50 mM at 30 mM of K_2PtCL_6.

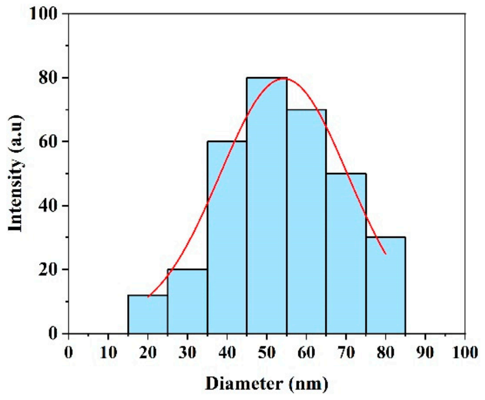

Figure S4. DLS analysis of Au@Pt at optimal condition.
